# Children's rights as law in Sweden–every health‐care encounter needs to meet the child's needs

**DOI:** 10.1111/hex.13060

**Published:** 2020-04-22

**Authors:** Sofia Sahlberg, Katarina Karlsson, Laura Darcy

**Affiliations:** ^1^ Department of Health Sciences Faculty of Caring Science, Work Life and Social Welfare University of Borås Borås Sweden

**Keywords:** children's rights, health‐care services, paediatric nursing, qualitative design, the United Nations Convention on the Rights of the Child, young children

## Abstract

**Background:**

In 2020, the United Nations Convention on the Rights of the Child (UNCRC) became law in Sweden. This puts extra demands on Swedish health care for children in need. This study aimed to investigate children's experiences and paediatric nurses' experiences of caring in accordance with the UNCRC.

**Methods:**

Interviews were conducted in 2019 with 10 children and 13 nurses at a paediatric clinic in western Sweden. Child and nurse data were analysed separately with qualitative content analysis. The results are presented as a synthesis of the combined analysis of both data sets.

**Results:**

Children did not always meet health‐care professionals with the necessary competence to care for them, and they were not always cared for in a child‐friendly environment. Even though nurses in paediatric care had the competence necessary to meet children's rights in health care, organizational issues made it challenging. Providing health care in accordance with the UNCRC principles required time and competence. Sufficient time to help children participate in their care and ensure that they feel secure was considered necessary—regardless of the health‐care context.

**Conclusion:**

Health‐care encounters without the necessary time or competence can affect children and future encounters negatively. Instruments to safeguard children's rights in health care need to be developed and implemented, such as a documentation system to make children's rights visible and/or UNCRC certification. Implementation of UNCRC principles in all health‐care situations for children as standardized care requires competence, involvement, strong leadership and economic support. Children's voices in research can assist in guiding care.

## 1 BACKGROUND

Children are entitled to the highest attainable health and the best possible health care when required.^1^ The majority of children and adolescents in Sweden encounter health‐care services at some point in childhood, during periods of ill health or during routine childhood check‐ups and immunizations. An ill child will require investigation and possible intervention, requiring various common medical investigations, such as needle‐related procedures, which can cause fear and pain.^2^ Fear and pain often intensify when children do not understand what is happening.^3^, ^4^, ^5^, ^6^, ^7^, ^8^ For children living with a long‐term illness where repeated hospital visits and procedures are common, fear and pain are part of the child's everyday life.^4^, ^9^, ^10^ Negative experiences of such procedures may affect the perspectives and experiences of the child during future health‐care encounters.^11^

The United Nations Convention on the Rights of the Child (UNCRC)^1^ was formulated in 1989 to safeguard the rights of children and young people, and as of 1 January 2020, it became Swedish law.^12^ This law aims to highlight children's rights and that these rights are considered in proceedings concerning children. The UNCRC consists of 54 articles, four of those articles are general principles which are helpful to interpret the rest of the convention (see Table 1).

**TABLE 1:** General principles of the UNCRC^1^

Article 2	Children shall be protected against all forms of discrimination
Article 3	The best interest of the child shall be the primary concern in all actions concerning children
Article 6	Children's inherent right to life, survival and development
Article 12	Children's rights to express their views, and their rights to have their views given due weight in accordance with their age and maturity

Of Sweden's approximately 10.2 million inhabitants,^13^ just over two million are under the age of 18,^14^ an age limit that separates children from adults.^1^ The rights of children in need of health care are regulated through various laws such as the Health Care Act,^15^ the Patient Act^16^ and now the UNCRC.^1^

Children are a vulnerable group and even more so when in need of health care. Human rights should be taken into account in every situation where children are in need of health care, at all levels including primary care, emergency care and in paediatric specialist care. Nurses caring for children need specific knowledge^17^ to maximize the benefits of care that can differ from caring for an adult. Parents and family play an important role in caring for children and in particular, providing security for the child. There is always a risk that children's needs and wishes are based on an adult's perspective of what is best for the child when the child is exposed to health care,^18^ also referred to as the adult having a child perspective.^19^ Young children's voices are often missing in research but should be heard.^2^, ^20^ The UNCRC becoming law in Sweden requires that nurses meet possible extra demands on health care when providing care for children. In order to gain an understanding of the difficulties that can arise in meeting the requirements of the UNCRC and how best address eventual difficulties, both children and nurses voices need to be heard. Therefore, the aim of this study was to investigate children and nurses in paediatric care experiences of caring in accordance with the UNCRC.

## 2 METHOD

The following table (Table 2) describes the research questions, based on the general articles from the UNCRC,^1^ which guided this study.

**TABLE 2:** Research questions

Article 2	Are children in health care treated equally?
Article 3	Is health care guided by what is best for the child?
Article 6	Are children receiving health care given opportunities to grow and develop?
Article 12	To what degree are children allowed to express and involve themselves in care?

### 2.1 Study design

This study had a qualitative, inductive design. It involves the synergy of separate analyses of interviews with children and nurses at a paediatric clinic in West Sweden during 2019.

### 2.2 Child interviews

#### 2.2.1 Settings

Child patients involved in the study came from across several paediatric areas: an outpatient department, an inpatient care unit, a day‐care unit and an emergency room.

#### 2.2.2 Participants

Eleven children were interviewed during April‐May and September‐October 2019. Two nurses working at the paediatric clinic asked a convenient sample of children and their parents, during appointments or on the phone, about participating in the study. Both parents and children were provided with verbal and written information—the written information had separate versions for parents and children. The parents also provided consent to the researcher to contact them for further information. The inclusion criteria were children between the ages of 4‐7 years with adequate verbal skills to undertake an interview. All children had experienced medical procedures such as needle or other invasive procedures. Parents gave written consent to participate in the study, and children gave their verbal assent—those with the developmental ability to sign their name for assent did so. The participating children were patients at the paediatric clinic for different medical conditions such as cancer, kidney disease, gastrostomy and vasculitis. In the data analysis, each child interview was named ‘C1’, ‘C2’, etc More detailed information is presented in Table 3.

**TABLE 3:** Child participant characteristics

Context	
Paediatric inpatient care unit	1
Paediatric outpatient care unit	3
Paediatric day‐care unit	5
Paediatric emergency room	1
Age of the children	
4	4
5	4
6	1
7	1
Gender	
Boy	6
Girl	4
Location for interview	
At the hospital	9
At home	1

#### 2.2.3 Data collection

Ten interviews, lasting for 9‐17 minutes with an average duration of 12 minutes, were included in the analysis. Ten interviews took place at the hospital, and one took place in one of the children's homes. One of the interviews was excluded from analysis due to the child changing their mind about participation. The interviewer, conducting every interview, was a nurse with master's degree and extensive experience in talking to children. To make the children feel comfortable and secure, parents were present during the interviews. This also facilitated establishing contact with the children.^2^ The interviewer began by playing with and talking to the children to make them comfortable with the interviewer.^2^ The parents present during the interviews provided a bridge between the interviewer and the children by helping their children understand the questions and sometimes explaining the children's answers to provide a deeper understanding.^20^, ^21^ Parents were allowed to interact with their children and the interviewer, by giving clues and examples, or clarifying the children's statements.^20^ Focus was on the child throughout the process, and information from parents was not included in the analysis. Questions based on Table 2 were asked in a way that children understood and complemented with follow‐up questions such as ‘tell me more’ and ‘what happened then?’

### 2.3 Nurse interviews

#### 2.3.1 Settings

Nurses at three paediatric primary health‐care units, a paediatric inpatient unit and a paediatric day‐care unit were included.

#### 2.3.2 Participants

Thirteen nurses were included in the study and interviewed during April‐May 2019. Eleven of the nurses were paediatric nurses with a master's degree, one was a registered nurse with a bachelor degree and one nurse was undergoing education to become a paediatric nurse. Information about the study was given verbally by the researcher during workplace meetings and via written material in March 2019. The inclusion criteria were to have experience from caring for children during different medical procedures such as needle or other invasive procedures. Interested nurses, via a contact nurse at each unit, gave permission to be contacted by the researcher for more information about the study. Those included in the study gave written consent to participate. The interviews were performed during working hours and lasted an average of 56 minutes. More detailed information is presented in Table 4.

**TABLE 4:** Nurse participant characteristics

Context	
Paediatric primary care units	9
Paediatric inpatient unit	1
Paediatric day‐care unit	3
Gender	
Women	13
Men	0
Education	
Paediatric specialist nurse	11
Registered nurse	2
Experience	
Numbers of years as a nurse	
Interval	8‐36
Average	22.9
Median	22
Group interviews	
Groups with two nurses	2
Group with four nurses	1
Group with five nurses	1

#### 2.3.3 Data collection

Interviews took place at the nurses' workplaces in order to maximize opportunities for the nurses to participate and to minimize interference with the daily work in the units. This meant that the nurses were interviewed together with colleagues from the same unit; see Table 4. Group interviews provide possibilities for interaction and deepened discussion from different viewpoints, and this is considered a viable method to reach the participants' experiences of a specific subject.^22^ All interviews were facilitated by the same researcher, and questions asked were based on Table 2. A second researcher kept notes and asked summarizing questions at the end of the interview. For the data analysis, transcripts were labelled ‘Group 1’, ‘Group 2’, etc

#### 2.3.4 Data analysis

**FIGURE 1:**
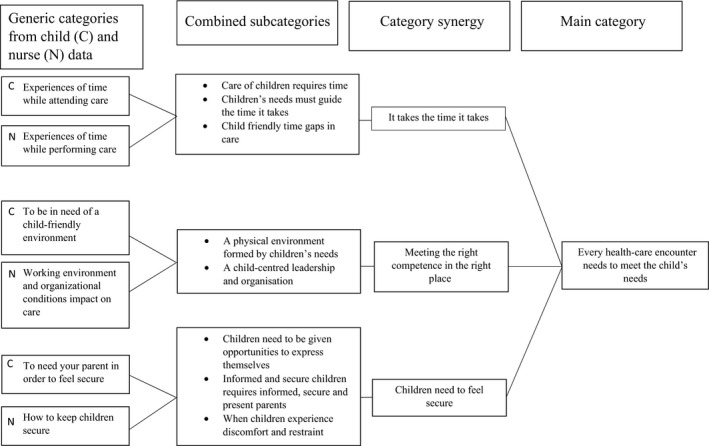
A synergy of child and nurse data

Interviews with children and nurses were audio‐recorded, identity removed and then transcribed verbatim. Both child and nurse data were analysed inductively with a qualitative content analysis.^23^ Initially, the interviews were read several times to gain a deeper understanding of the content of data. Narrations that concerned the study aim were first highlighted and secondly condensed to codes. In step 3, codes were freely sorted into categories based on content. In the fourth step, through discussions in the research group, subcategories were formed that described the content of the data. In step 5, through discussion within the research group, subcategories were merged to form generic categories. Steps 1‐5 were performed for each data set separately. When those steps of analysis were made, a complementary step as described by Darcy et al^21^ followed, whereby a synthesis based on combined analysis of both subcategories and generic categories from both the children's data and nurses' data was made. Similarities and differences in children's data and nurses' data were sought, and in discussion with the research group, the synthesis of both groups of data resulted in a synergy of combined subcategories, generic categories and one main category (see Figure 1).

### 2.4 Ethical considerations

The four ethical principles of respect for autonomy, beneficence, non‐maleficence and justice in accordance with the Helsinki Declaration were considered during the research process.^24^ The study received ethical approval by the Regional Ethical Review Board in Gothenburg, Sweden (dnr 1025‐18). Parents and children were given information about the study by nurses at the paediatric units and asked whether the researcher could contact them to provide further information. Interested parents received detailed verbal and written information, and children received verbal information with pictorial support. Both parents gave their written consent and children their verbal assent to participate in the study.^25^ Confidentiality was assured as well as the children's right and parents' right to withdraw their consent during the study.

## 3 RESULTS

### 3.1 Every health‐care encounter needs to meet the child's needs

Attending health‐care services as a child, or providing health care to children as a professional, is a process experienced to take considerable time. Sufficient time was needed to make children participate and feel secure. Children's experiences of time‐consuming visits in health care also had to be considered while planning care for children. Children needed to meet health‐care professionals with special competence in every health‐care meeting. All in all, according to nurses, this is needed to be considered even from organizational level to keep children feeling secure and participating in their health‐care encounters.

#### 3.1.1 It takes the time it takes

Both children and nurses pointed out that providing and experiencing child‐friendly care and increasing children's participation in their care took time. Visits and procedures in health care were time‐consuming from the children's perspective. Time spent waiting was, according to children, easier to endure if filled with meaningful activities.

##### Care of children requires time

Care of children required time, time to increase participation, and make children feel secure. Children wanted playful nurses who could make medical procedures and hospital visits easier. Both children and nurses told how diversion, having a sense of humour and applying a gentle approach offered comfort to children and their parents; ‘*Tell me something funny – sing a song or something!’ [Girl, five years old, C7].*

Children found it important to be able to influence medical procedures, and this required creativity on the nurse's part. However, lack of time was detrimental to creativity, and the ability to find strategies to involve children in their care experienced a lack of time; ‘*…because the first thing you lose when you are stressed for time is your creativity…’ [Nurse, group 4].*

Nurses experienced feelings of stress and frustration from the fact that they would be able to provide more optimal care under more favourable conditions. Nurses pointed out that time invested in preparing children for procedures often pay off in the long run by facilitating future health‐care encounters.

##### Children's needs must guide the time it takes

Children's needs must guide the time it takes when providing care for children. Time‐consuming hospital visits or procedures were experienced as tiresome for children; ‘*I don't like it when the visit takes too long!’ [Girl, four years old, C10].*

When planning care for children, nurses were of the opinion that what is best for the child should guide procedures, rather than the parents or the organizations. Nurses avoided scheduling several appointments or examinations after another and often booked in new times for children to leave samples, etc Nurses experienced that this sometimes conflicted with parent's interests, such as not having to take extra time off from work. Procedures often required a certain amount of time and could not be rushed if children were to be participants in their care. Therefore, nurses reported that they rather rescheduled (when possible) than expose the child to non‐optimal health‐care situations.

##### Child‐friendly time gaps in care

Child‐friendly time gaps in care were important to reduce feelings of boredom and to relieve feelings of homesickness, according to children. How these time gaps were filled was particularly important for children who were bound to a certain room or bed‐bound. Children who frequently attended health‐care services described how time‐consuming treatments left them longing to take part in everyday activities, such as going to school, being with friends or engaging in different hobbies. The play department became a free zone where they could engage in activities that created meaning for them; ‘*I like clowns! And the play department.’[Boy, five years old, C4].*

Nurses reported that child‐friendly time gaps and meaningful activities made children feel more at home while visiting the hospital. They could also be used to prepare children before medical procedures or to process difficult experiences after procedures or examinations.

#### 3.1.2 Meeting the right competence in the right place

Both children and nurses pointed out that children need an environment suited to their needs, where they feel at home and where they can be alone with their parents and their nurse. Nurses emphasized how children also need to meet health‐care professionals with competence in providing health care to children, regardless of which level of care they use. Medical or organizational constraints were experienced by nurses as sometimes overshadowing nursing competence and children's needs, and instruments to safeguard children's rights in health care are missing.

##### A physical environment formed by children's needs

A physical environment formed by children's needs is important to make children feel secure. Both children and nurses highlighted how challenging caring situations, such as blood sampling, can put extra demands on the physical environment. The possibility of being alone and undisturbed in a safe place with your nurse and your parents was considered important—this safe place could even be outdoors: ‘*It may be better to move [blood sampling] outside… […] There is a lot of people indoors watching me…’ [Girl, four years old, C5].*

Nurses reported that it was not uncommon for children to develop fears related to sampling situations. The paediatric clinic offered extra support for those children through an educative ‘needle school’ where children could treat their fears. Children in needle school often had developed fears after needle‐related procedures in other units, such as a primary care setting or the emergency room. The environment on those units were told to often be adult‐focused, stressful and children had to undergo needle‐related procedures in the same room at the same time as others. Nurses believed that encouraging child‐focused care everywhere in health care was an important strategy to prevent children developing needle phobia; ‘*If we succeed in making these brief contacts [with healthcare professionals] positive experiences the first time […] it gets so much easier for everyone.’ [Nurse, group 1].*

##### A child‐centred leadership and organization

A child‐centred leadership and organization must provide instruments that make children's rights visible and how to implement them in daily work. Sometimes nurses experienced how medical views and logistical issues tended to be decisive and overshadow children's needs; ‘*Who takes children's best into consideration, organizationally?’ [Nurse, group 3].*

Sometimes the nurses expressed difficulties in highlighting their professional and specialist knowledge. Instruments, such as the documentation system, were described as lacking possibilities to document important nursing aspects in health care for children such as communication and participation. Keywords were medically focused and designed for medical specialists. Places to document how the child attended to their care, if they participated or not, if they needed to be held or convinced to endure a procedure, were often missing.

#### 3.1.3 Children need to feel secure

Children actively participating in their own care were only found possible if the children were well‐informed and felt secure. Nurses found parents as an important partner in achieving this goal. Nurses considered children's viewpoints when providing health care, but in spite of this children can experience discomfort or even restraint while undergoing care procedures.

##### Children need to be given opportunities to express themselves

Children need to be given opportunities to express themselves. It was essential for children to feel confident with their nurse in order to communicate their feelings when something is challenging or frightening; ‘*My nurses usually ask me what I think and stuff…’ [Girl, four years old, C10].*

Nurses experienced that emergencies often meant fewer possibilities for children to express themselves and communication became more parent‐centred. Even so, it was important to give children possibilities to express themselves in acute situations, when possible.

Children were encouraged to express their thoughts and wishes on aspects of care, but medical procedures had to be followed through anyway. One nurse explained how to be honest with a child before such a procedure; ‘*Yes, we know that you don't want to do this and that you find this scary, but sometimes you have to do things you don't want to […] You are allowed to get sad, and it does hurt…’ [Nurse, group 3].*

Nurses found parents essential to help their children communicate with health‐care professionals, particularly when children were scared and not willing to participate. When communicating with children with special needs, parents were even more important to help children express their feelings. Parents sometimes left no room for their child to speak, particularly when parents themselves had unanswered worries or fears. This could hinder children's possibilities to express themselves.

##### Informed and secure children require informed, secure and present parents

Informed and secure children require informed and secure parents. Both children and nurses explained how closeness to parent(s) is important for children to feel secure and make different medical procedures easier to endure; ‘*It gets easier when I hold mummy's hand…’ [Girl, four years old, C8].*

Children expressed that they were generally informed about what is going to happen prior to their health‐care visits through their parents. Nurses explained that parents must be well informed in order to give necessary information in a way suitable for the child. Some children told that they talk about the visit well in advance, while others were so used to the procedure that they only needed to know what would happen and required no further information. Sometimes the families had received picture material before the visit. Knowing what is about to happen was very important information for children. This was considered even more important when offering health care to children with special needs, where parents are important as partners to professionals to create a caring situation and good contact with the child.

##### When children experience discomfort and restraint

Children described experiences of procedures that are challenging and uncomfortable. Sometimes procedures, tests, or treatments had to be done at a certain time or occasion with little input from children. In those situations, nurses tried to make the best of the situation by giving children opportunities to make smaller decisions related to the procedures such as choosing the colour of the bandage or express their fears as they wish; ‘*So, sometimes it can get bad when I get stung […] then I cry out loud like this…’ [screams][Boy, four years old, C1].*

Physical restraint was sometimes considered necessary by nurses when children were not willing to participate in a procedure that had to be undergone, to avoid injuries to the child. Restraint could consist of holding a child's arm under simultaneous distraction and with the policy to stop if it did not work. The general agreement amongst nurses was that only one professional was allowed to hold the child in such situations. In less acute situations, nurses could give children and parents the choice to postpone a test or an examination.

## 4 DISCUSSION

### 4.1 Discussion of the method

This is a small but insightful study. The study was performed before the UNCRC became law in Sweden, as even small, qualitative studies can help us guide and plan.^20^ The study includes data from interviews with children and group interviews with nurses. Collecting data from more than one perspective gains a broader understanding of how children experience receiving health care, how nurses experience providing care, and how their experiences correspond to the fundamental values in the UNCRC. The data analysis was examined through a synergy of data from both children and nurses, to find similarities and differences in children and nurses' narrations. While not unproblematic, the process of synergizing two data sets has been previously performed successfully^21^ and adds a broader perspective, in this study an understanding of similarities and differences in children's experiences and nurses' experiences of children's rights in paediatric care. The synergy of data was sometimes challenging. Nurses reasoned extensively about organizational issues, issues that children naturally did not consider. The aspect of time was prominent in both children's interviews and nurses' interviews—but from different perspectives—and helped to gain an understanding of how time affects both children's experiences and nurses' experiences of care.

The analysis process obtained trustworthiness by frequent discussions in the research group concerning the content of data in the different subcategories and generic categories. To illustrate research findings,^26^, ^27^ citations from the interviews were used.

Hearing children talk about health‐care experiences is not common in research, and making their voices heard is essential to increase understanding of how to implement UNCRC values in health care. Interviewing children can be difficult, and using parents to help was found valuable to make the children feel safe and to establish contact with them.^21^ The child was the focus, not the adult. Even so, parents provided an important bridge between the researcher and these young children. The research group has extensive experiences of interviewing children; even so, it is a challenging task. It was sometimes a short window of attention we had from the attending children and challenging to keep the focus on the subject for the interviews. With some children, playing during the interview was necessary to keep them interested in interaction with the researcher.^2^, ^21^, ^27^ However, it is sometimes difficult to balance the focus on interview questions and playing with the child. Children associate rapidly, and to make them willing to interact with the researcher, who was unknown to them, it was important to be compliant to their choice of topic.

Our ambition was to have focus group interviews with mixed groups of nurses from the various departments as focus groups may offer possibilities for common reflection and discussion on the research questions.^22^ Due to difficulties in recruiting enough nurses for focus group interviews, interviews were conducted in smaller groups and in each separate clinic or department. The number of participants in each group and the local settings were chosen in consultation with the nurses in order not to interrupt clinical practice. In smaller groups, each person could easily make one's voice heard and it was fairer for the group moderator to handle.^22^ The groups got quite homogeneous, though there were members from the same unit and with similar backgrounds. McLafferty^27^ has found that homogeneous groups often work better than heterogeneous groups.

Children in the study were patients at a paediatric clinic, and this was where they experienced many health‐care‐related issues. However, it most likely that the children's narratives about what is essential, what is difficult, and what makes it easier for them while receiving health care apply to other health‐care units as well. It is important to clarify that this study was conducted the year before the UNCRC became a law in Sweden. Work with increasing the UNCRC values in Swedish health care is on‐going, but it is too soon to evaluate the outcome from this work.

### 4.2 Discussion of results

Every care encounter needs to meet the child's needs to achieve the principles of the UNCRC. Leading principles is about treating all children equally (Article 2), to make children's best be governed in all decisions concerning children (Article 3), children's inherent rights to life, survival and development (Article 6) and children's rights to express their views and have it taken into account (Article 12). Positive experiences when receiving health care are necessary to increase sustainability in health care for children. Sustainability is necessary for every individual child as well, where anxiety or stress does not just come from non‐optimal health‐care situations, but also come from health‐care providers themselves—scared children who feel insecure demand extra efforts from those caring for them. Proper communication prevents feelings of stress and results in positive experiences from health‐care encounters.^28^ Every encounter children have with health‐care counts, independent of the level of care needed.

### 4.3 It takes the time it takes

Paediatric nurses had the competence to offer health care, according to the UNCRC values. They knew how to do it, but they lacked the working conditions necessary to work according to their competence. Lack of time was one aspect that prevented them in some situations, not least in more acute situations where there was an agreement that there are challenges to children expressing themselves and participating in their care. Increasing participation is important to ensure growth and development. Furthermore, a stressful working environment inhibits nurses' capability to stay creative and successfully individualize care for each child. A sufficient amount of time is necessary to make children and their parents well‐informed and prepared for different medical procedures.^29^ One can conjecture, when it comes to children with disabilities and children in need of an interpreter, that lack of time also may lead to decreased effective communication.^29^, ^30^, ^31^ Lack of time in health care for children would then tend to risk falling short of Article 2 in the convention,^1^ which states that all articles in the convention are for all children and that discrimination of any kind, as from language or disability, shall be countered.^1^ Even Article 23, which states disabled children's rights to obtain conditions that ensure dignity and enable self‐reliance and active participation in the community,^1^ seems to be a challenge when caregivers do not have a sufficient amount of time to create optimal health‐care encounters.

### 4.4 Meeting the right competence in the right place

Children need to meet professionals with adequate competence in giving care to children in an environment suited to children's needs.^32^ Children who experience unsecure encounters due to lack of expertise in communication with children, or due to an environment experienced as unsecure, are at risk of developing fears and anxiety that may affect future health‐care encounters. Paediatric care is largely designed for children's needs in contrast to many other health‐care services such as emergency rooms^33^ or primary care. Professionals in those units are unlikely to have the skills or possibilities to assess effective communication,^34^ which is necessary to achieve children's rights to express their opinion according to Article 12.^1^ The results of this study indicate that children's ability to express their opinions and take an active part in health care is reduced in acute care emergency care situations. The results also show that facilities outside of paediatric care seem to be less prepared to meet the UNCRC as law.

Swedish law established in the Patient Act^15^ 2015 highlights the importance of clear, developmentally appropriate care information to children. It requires specific competence to customize effective communication with children according to their age, level of maturity or any disabilities and make it possible for children to express their opinions. Participation is not about the child wanting to participate in all care or giving consent to all efforts, but to try to find solutions that work as well as involving children to a great extent.^34^ Dialogue with the child is essential to encourage child participation in care. Interviews with children show that a trustful relationship with health‐care professionals is important^35^ and that they want the possibility to influence their care.^32^

### 4.5 Children need to feel secure

Children need their parents to keep them well informed and to feel secure when receiving health care.^16^, ^29^ According to Article 9,^1^ children shall not be separated from their parents. While caring for children, we also care for their parents. However, there is always a risk that children's needs can be overshadowed by parents' needs or opinions and pose a risk to Article 3. A child‐centred perspective gives children a voice when receiving health care. The individual child is considered as the central subject but in close relation with and dependent on its family. In this way, autonomy and the child's competence are supported.^36^ With this in mind, self‐efficacy, as described by Banduras^37^ as trust in one's abilities to cope with a specific task in one particular situation, may be increased, and health‐care situations can contribute to the growth and development of children, in accordance with Article 6.^1^

Restraint occurs in health care for children.^38^, ^39^ From the viewpoint of children, restraint is never acceptable^40^ and may pose a risk to growth and development. Restraint is also complicated in the light of Article 19, which states that children be protected from physical violence and abuse.^1^ Sufficient time and competence in how to make children participatory seem to be essential for avoiding less than optimal care and in extreme forms even restraint.

Nurses in paediatric care in Sweden have knowledge and competence in children's rights. However, aspects such as a non‐child‐friendly environment, lack of time and experiencing stress may result in less than optimal care. Children sometimes experience medical procedures in care as traumatizing, often due to health‐care encounters that treat children inhumanely. Use of an ethical framework may be helpful in order to offer humane care to children by seeing the child as a unique individual.^40^ Children's perspectives need to be taken into account, and children need help to create context and comprehensibility while receiving health care.^41^ True child‐centred care requires involvement, strong leadership and economic support.^36^ In summary, there seems to be a gap between organizational values and decisions and the extent they are guided by what is best for the child. Nurses and children's experiences shared in this study show that the general principles of the UNCRC seem to be endangered in various situations and that more attention needs to be directed to those principles when planning and performing care to children in all health‐care situations. Research including children's voices may increase professional knowledge and insight into how to make health care more humane to children and guide care.

## 5 CLINICAL IMPLICATIONS

Nurses in this study expressed how it is challenging to claim nursing competence, when other values, such as medicinal and organizational values, seem to be dominant. Nursing interventions to make UNCRC values visible, for example in the documentation system, are lacking. A documentation system with extended opportunities to document children's participation in care would also provide opportunities to document situations where restraint has occurred.^38^ Tools to safeguard children's rights in health care are required. Streuli et al^42^ present a checklist based on the articles in the convention to protect children's rights in health care, and this may be implementable in Swedish health care. Interventions in Swedish health care, such as education to health‐care professionals in children's rights based on the UNCRC as well as education of ‘children right agents’, have been initiated.^43^, ^44^ It is too soon to tell if those interventions are sufficient to safeguard children's needs when encountering health care. Perhaps the development and implementation of a UNCRC certification of health‐care services would assist in the implementation of the UNCRC values in all situations concerning children in health care. This would help to ensure secure encounters with children in health care.

## 6 CONCLUSIONS

This study shows that nurses in paediatric care have the competence necessary to meet children's rights in health care. However, organizational issues make it challenging to work according to children's rights. Organizationally, Article 3^1^ tends not to be taken into sufficient consideration in all decisions that affect children in Swedish health care. It is of the utmost importance that politicians and health‐care management acquire necessary knowledge of children's needs and rights when making decisions relating to health‐care procedure and policy. In the absence of this specific knowledge, health care to children is not sustainable. Paediatric nurses have training in communication with children. This specific competence is often missing in units outside of paediatric care, and consequently, children may experience negative encounters. Children need to be cared for at health‐care units with specialist paediatric competence. Though the result shows that units outside the paediatric clinic more often lack expertise in offering health care according to UNCRC values, further research with professionals and children in those contexts is required. Nurses working in paediatric care lack specific instruments to achieve UNCRC values in daily health‐care situations. Specific nursing competence and instruments that increase children's participation are required to take children's rights into account in all health‐care encounters.

## CONFLICT OF INTEREST

None.

## AUTHOR CONTRIBUTIONS

KK and LD designed the study. SS, KK and LD collected and analysed the data and prepared the manuscript.

## Data Availability

Data are available on request.

## References

[1] United Nations . Conventions on the Rights of the Child. https://www.ohchr.org/en/professionalinterest/pages/UNCRC.aspx Published November 20, 1989. Accessed January 2, 2020.

[2] Karlsson K , Dalheim Englund A‐C , Enskär K , Nyström M , Rydström I . Experiencing support during needle‐related medical procedures: A hermeneutic study with young children (3–7 years). J Paediatr Nurs. 2016;31(6):667‐677. 10.1016/j.pedn.2016.06.004 27426015

[3] Kettwich SC , Sibbitt WL , Brandt JR , Johnson CR , Wong CS , Bankhurst AD . Needle phobia and stress‐reducing medical devices in paediatric and adult chemotherapy patients. J Paediatr Oncol Nurs. 2007;24(1):20‐28. 10.1177/1043454206296023 17185398

[4] Salmela M , Salanterä S , Aronen ET . Child‐reported hospital fears in 4 to 6‐year‐old children. Paediatr Nurs. 2009;35(5):269‐276. 19916342

[5] Taddio A , Chambers CT , Halperin SA , et al. Inadequate pain management during routine childhood immunizations: The nerve of it. Clin Ther. 2009;31:152‐167. 10.1016/j.clinthera.2009.07.022 19781434

[6] Salmela M , Aronen ET , Salanterä S . The experience of hospital‐related fears of 4‐ to 6‐ year‐old children. Child Care Health Dev. 2011;37(5):719‐726. 21143264 10.1111/j.1365-2214.2010.01171.x

[7] Taddio A , Ilersich AF , Ilersich AN , Wells J . From the mouth of babes: Getting vaccinated doesn’t have to hurt. Can J Infect Dis Med Microbiol. 2014;25(4):196‐200. 25285123 10.1155/2014/470261 PMC4173939

[8] McMurtry CM , Riddell RP , Taddio A , et al. Far from “just a poke”: common painful needle procedures and the development of needle fear. Clin J Pain. 2015;31(10):S3. 26352920 10.1097/AJP.0000000000000272 PMC4900413

[9] Bird L , McMurtry CM . Fear in paediatric acute pain: role and measurement. Pain Manage. 2012;2(6):527‐529. 10.2217/pmt.12.56 24645882

[10] Aydin D , Sahiner NC , Ciftçi EK . Comparision of the effectiveness of three different methods in decreasing pain during venipuncture in children: ball squeezing, balloon inflating and distraction cards. J Clin Nurs. 2016;25(15–16):2328‐2335. 27112434 10.1111/jocn.13321

[11] Nunns M , Mayhew D , Ford T , et al. Effectiveness of nonpharmacological interventions to reduce procedural anxiety in children and adolescents undergoing treatment for cancer: a systematic review and meta‐analysis. Psycho‐Oncol. 2018;27(8):1889‐1899. 10.1002/pon.4749 29714037

[12] UN Convention on the rights of the child to become law in Sweden .Swedish Parliament website. https://www.riksdagen.se/en/news/2018/jun/18/un‐convention‐on‐the‐rights‐of‐the‐child‐to‐become‐law‐in‐sweden/ Accessed January 2, 2020.

[13] Befolkningsstatistik . [Demography]. Statistics Sweden website. https://www.scb.se/hitta‐statistik/statistik‐efter‐amne/befolkning/befolkningens‐sammansattning/befolkningsstatistik/. Accessed January 2, 2020.

[14] Sveriges befolkningspyramid . [The population pyramid of Sweden.] Statistics Sweden website. https://www.scb.se/hitta‐statistik/sverige‐i‐siffror/manniskorna‐i‐sverige/sveriges‐befolkningspyramid/. Accessed January 20, 2020.

[15] SFS 1982:763 . Halso‐ och sjukvardslagen. [SFS 1982:763. The health and medical services act.] The Swedish Parliament. https://www.riksdagen.se/sv/dokument‐lagar/dokument/svensk‐forfattningssamling/halso–och‐sjukvardslag_sfs‐2017‐. Accessed February 13, 2020.

[16] SFS 2014:821 . Patientlagen. [SFS 2014:821. The patient act.] The Swedish Parliament. https://www.riksdagen.se/sv/dokument‐lagar/dokument/svensk‐forfattningssamling/patientlag‐2014821_sfs‐2014‐821. Accessed February 13, 2020.

[17] Karlsson K , Rydström I , Enskär K , Dalheim Englund A‐C . Nurses’ perspectives on supporting children during needle‐related medical procedures. Int J Qual Stud Health Well‐being. 2014;9(1):1‐11. 10.3402/qhw.v9.23063 PMC3955765 24646473

[18] Coyne I , Hallstrom I , Soderback M . Reframing the focus from a family‐centred to a child‐centred care approach for children’s healthcare. J Child Health Care. 2016;20(4):494‐502. 27141084 10.1177/1367493516642744

[19] Sommer D , Pramling Samuelsson I , Hundeide K . Barnperspektiv och barnens perspektiv i teori och praktik. [A child perspective and children’s perspective in theory and practice.]. Stockholm: Liber AB; 2011.

[20] Darcy L , Knutsson S , Huus K , Enskar K . The everyday life of the young child shortly after receiving a cancer diagnosis, from both children's and parent's perspectives. Cancer Nurs. 2014;37(6):445‐456. 24406380 10.1097/NCC.0000000000000114

[21] Darcy L , Björk M , Enskär K , Knutsson S . The process of striving for an ordinary, everyday life, in young children living with cancer, at six months and one year post diagnosis. Eur J Oncol Nurs. 2014;18(6):605‐612. 24997519 10.1016/j.ejon.2014.06.006

[22] Trost J . Kvalitativa intervjuer. [Qualitative interviews.]. Lund: Studentlitteratur AB; 2010.

[23] Elo S , Kyngäs H . The qualitative content analysis process. J Adv Nurs. 2008;62(1):107‐115. 18352969 10.1111/j.1365-2648.2007.04569.x

[24] World Medical Association . WMA Declaration of Helsinki – ethical principles for medical research involving human subjects. https://www.wma.net/policies‐post/wma‐declaration‐of‐helsinki‐ethical‐principles‐for‐medical‐research‐involving‐human‐subjects/ Published July 9, 2018. Accessed January 3, 2020.

[25] Neill SJ . Research with children: a critical review of the guidelines. J Child Health Care. 2005;9(1):46‐58. 15684439 10.1177/1367493505049646

[26] Polit DF , Beck CT . Nursing Research: Generating and Assessing Evidence for Nursing Practice. Philadelphia, PA: LWW Publications; 2008.

[27] McLafferty I . Focus groups interviews as a data collecting strategy. J Adv Nurs. 2004;48(2):187‐194. 15369499 10.1111/j.1365-2648.2004.03186.x

[28] World Health Organization . Standards for improving the quality of care for children and young adolescents in health facilities. 2018 https://apps.who.int/iris/bitstream/handle/10665/272346/9789241565554‐eng.pdf?ua=1 Published 2018. Accessed January 31, 2020.

[29] Grahn M , Olsson E , Edwinson MM . Interactions between children and paediatric nurses at the emergency department: a Swedish interview study. J Paediatr Nurs. 2016;31:284‐292. 10.1016/j.pedn.2015.11.016 26992940

[30] Sharkey S , Lloyd C , Tomlinson R , et al. Communicating with disabled children when inpatients: barriers and facilitators identified by parents and professionals in a qualitative study. Health Expect. 2014;19(3):738‐750. 25156078 10.1111/hex.12254 PMC5055242

[31] Williams A , Oulton K , Sell D , Wray J . Healthcare professional and interpreter perspectives on working with and caring for non‐English speaking families in a tertiary paediatric healthcare setting. Ethnicity and Health. 2018;23(7):767‐780. 28277020 10.1080/13557858.2017.1294662

[32] K leye I , Karlsson K , Hedén L , Sundler AJ . DarcyLChildren’s individual voices are required for adequate management of fear and pain during hospital care and treatment. Scandinavian Journal of Caring Science. (Accepted March2020)​. 10.1111/scs.12865 32363693

[33] Janhunen K , Kankkunen P , Kvist T . Nursing staff’s perceptions of quality of care for children in emergency departments – high respect, low resources. J Paediatr Nurs. 2017;37:10‐15. 10.1016/j.pedn.2017.08.029 28887048

[34] The National Board of Health and Welfare . Bedöma barns mognad för delaktighet. Kunskapsstöd för socialtjänsten, hälso‐ och sjukvården samt tandvården. [Assess children’s maturity for participation. Knowledge support for social services, health care and dental care.] https://www.socialstyrelsen.se/globalassets/sharepoint‐dokument/artikelkatalog/kunskapsstod/2015‐12‐22.pdf Published December 22, 2015. Accessed January 12, 2020.

[35] The National Board of Health and Welfare . Att samtala med barn. Kunskapsstöd för socialtjänsten, hälso‐ och sjukvården samt tandvården. [To talk to children. Knowledge support for social services, health care and dental care. https://www.socialstyrelsen.se/globalassets/sharepoint‐dokument/artikelkatalog/kunskapsstod/2018‐11‐14.pdf Published December 3, 2018. Accessed January 12, 2020.

[36] Coyne I , Hallstrom I , Soderback M . Centeredness in healthcare: a concept synthesis of family‐centered care, person‐centered care and child‐centered care. J Paediatr Nurs. 2018;42:45‐56. 10.1016/j.pedn.2018.07.001 30219299

[37] Banduras A . Self‐efficacy: toward a unifying theory of behavioral change. Psychol Rev. 1977;84(2):191‐215. 847061 10.1037//0033-295x.84.2.191

[38] Kangasniemi M , Papinaho O , Korhonen A . Nurses' perceptions of the use of restraint in paediatric somatic care. Nursing Ethics. 2014;21(5):605‐620. 10.1177/0969733013513214 24493711

[39] Karlsson K . “I’m afraid, I want my mommy” : Younger children's, parents', and nurses' lived experiences of needle procedures in health care. Jönköping: University of Jönköping; 2015.

[40] Darcy L , Karlsson K . Humanising care for sick children in hospital: ‐are we ready to meet the demands of The Convention on Human Rights of the Child (CHRC)? In: Athens: 4th PNAE Congress on Paediatric Nursing. 2018.

[41] Karlsson K , Galvin K , Darcy L . Medical procedures in children using a conceptual framework that keeps a focus on human dimensions of care – a discussion paper. Int J Qual Stud Health Wellbeing. 2019;14(1):1‐14. 10.1080/17482631.2019.1675354 PMC6807864 31621530

[42] Streuli JC , Michel M , Vayena E . Children's rights in paediatrics. Eur J Paediatr. 2011;170(1):9‐14. 10.1007/s00431-010-1205-8 20461530

[43] Vardforbundet . Sa starks barns ratt i varden. [This will strengthen children’s rights in healthcare]. https://www.vardfokus.se/webbnyheter/2020/januari/nu‐starks‐barns‐ratt‐i‐varden/ Published January 2020. Accessed February 13, 2020.

[44] Johansson L . Barnkonventionen blir lag 2020 – så här förbereder vi oss i Västra Götalandsregionen. [The Convention on the Rights of the Child becomes law in 2020 – this is how we prepare in Region Vastra Gotaland]. Barnbladet. 2019;5:30‐31.

